# Extracellular Vesicle microRNAs Contribute to the Osteogenic Inhibition of Mesenchymal Stem Cells in Multiple Myeloma

**DOI:** 10.3390/cancers12020449

**Published:** 2020-02-14

**Authors:** Stefania Raimondo, Ornella Urzì, Alice Conigliaro, Giosuè Lo Bosco, Sofia Parisi, Melania Carlisi, Sergio Siragusa, Lavinia Raimondi, Angela De Luca, Gianluca Giavaresi, Riccardo Alessandro

**Affiliations:** 1 Department of Biomedicine, Neuroscience and Advanced Diagnostics (Bi.N.D.), Section of Biology and Genetics, University of Palermo, 90133 Palermo, Italy; stefania.raimondo@unipa.it (S.R.); ornella.urzi@gmail.com (O.U.); alice.conigliaro@unipa.it (A.C.); sofia.parisi91@gmail.com (S.P.); 2 Department of Mathematics and Computer Science, University of Palermo, 90133 Palermo, Italy; giosue.lobosco@unipa.it; 3 Department of Sciences for technological innovation, Euro-Mediterranean Institute of Science and Technology, 90133 Palermo, Italy; 4 Department of Health Promotion, Mother and Child Care, Internal Medicine and Medical Specialties (ProMISE), Haematology Unit, University of Palermo, 90133 Palermo, Italy; melania.carlisi@unipa.it (M.C.); sergio.siragusa@unipa.it (S.S.); 5 IRCCS Istituto Ortopedico Rizzoli, 40136 Bologna, Italy; lavinia.raimondi@ior.it (L.R.); angela.deluca@ior.it (A.D.L.); gianluca.giavaresi@ior.it (G.G.)

**Keywords:** extracellular vesicles (EVs), exosomes, multiple myeloma (MM), bone disease, microRNAs, osteogenic differentiation, transcription factor sp1

## Abstract

Osteolytic bone disease is the major complication associated with the progression of multiple myeloma (MM). Recently, extracellular vesicles (EVs) have emerged as mediators of MM-associated bone disease by inhibiting the osteogenic differentiation of human mesenchymal stem cells (hMSCs). Here, we investigated a correlation between the EV-mediated osteogenic inhibition and MM vesicle content, focusing on miRNAs. By the use of a MicroRNA Card, we identified a pool of miRNAs, highly expressed in EVs, from MM cell line (MM1.S EVs), expression of which was confirmed in EVs from bone marrow (BM) plasma of patients affected by smoldering myeloma (SMM) and MM. Notably, we found that miR-129-5p, which targets different osteoblast (OBs) differentiation markers, is enriched in MM-EVs compared to SMM-EVs, thus suggesting a selective packaging correlated with pathological grade. We found that miR-129-5p can be transported to hMSCs by MM-EVs and, by the use of miRNA mimics, we investigated its role in recipient cells. Our data demonstrated that the increase of miR-129-5p levels in hMSCs under osteoblastic differentiation stimuli inhibited the expression of the transcription factor Sp1, previously described as a positive modulator of osteoblastic differentiation, and of its target the Alkaline phosphatase (ALPL), thus identifying miR-129-5p among the players of vesicle-mediated bone disease.

## 1. Introduction

Multiple myeloma (MM) represents the second most prevalent hematologic malignancy [1], is classified in a heterogeneous group of diseases, plasma cellular dyscrasias, characterized by the monoclonal expansion of bone marrow (BM) plasma cells that release a monoclonal immunoglobulin, called M component or paraprotein. The occurrence of MM is consistently preceded by two precursor states: a pre-cancerous condition called gammopathy of undetermined significance (MGUS) and the asymptomatic clonal plasma cell disorder called smoldering myeloma (SMM). SMM is distinguished from MGUS by a much higher risk of progression to MM [2].

The major complication, associated with MM disease progression, which is not observed in the other two conditions, is the bone disease. In fact, around 80% of patients develop osteolytic bone lesions that lead to fractures and bone pain [3]. The occurrence of osteolytic bone disease is the consequence of the alteration of the physiological balance between the activity of bone-resorbing osteoclasts (OCs) and bone-forming osteoblasts (OBs) that results in the concomitant activation of osteoclastogenesis and inhibition of osteoblastogenesis [4,5]. A better understanding of the underlying causes of bone disruption in patients affected by MM is needed to develop new targeted therapeutic strategies aimed at inhibiting osteolysis and stimulating OB differentiation. It has been extensively reported that MM cells are able to induce the remodeling of the bone marrow stromal tissue to promote their growth and survival. For this purpose, the bidirectional crosstalk between MM cells and Bone marrow stromal cells (BMSCs) is crucial [6].BMSC differentiation is regulated by a number of cytokines as well as miRNAs [7,8]. To note, several miRNAs can exhibit inhibitory roles in osteogenesis by directly or indirectly targeting key osteogenic factors [9]. In order to further investigate the multiple mechanisms adopted by MM cells to influence the tumor microenvironment, in the last years, we and other research groups have been working on understanding if MM-extracellular vesicles(EVs) can affect the balance between OC and OB functions.

EVs are a heterogeneous population of membrane-bound vesicles, released by almost all cell types, differing in sizes, secretory pathways, and content [10,11]. EVs have a sophisticated composition, including lipids, proteins, DNA, coding and non-coding RNA species, and, through different mechanisms, can interact with target cells, leading to phenotypic changes of recipient cells. For all these reasons, they are considered mediators of intercellular communication also in the tumor microenvironment. Numerous observations indicate that EVs play a crucial role in MM pathogenesis [12,13] by affecting endothelial cell functions [14,15,16,17], enhancing tumor cell proliferation [18], and promoting immunosuppression [14,19]. In addition, EVs that contain miRNAs may be an important means of cell–cell communication within the MM tumor microenvironment [20]. Recent studies have shown that MM cell-derived EVs play a relevant functional role in activating osteoclast differentiation [21,22,23] and that the presence of the EGFR ligand amphiregulin is partially responsible for OC activation [21]. Interestingly, increasing evidence reported that MM EVs are also effective in inhibiting osteogenesis, in particular by blocking the osteoblastic differentiation of mesenchymal cells [21,23,24,25,26,27]. In particular, the decrease in ALPL levels, both at the transcript and protein levels, was largely correlated with MM-EVs mediated osteogenic inhibition [21,23,24]. We have previously demonstrated that MM1.S-derived EVs led to a reduced expression of OB differentiation markers (ALPL, OCN, Col1A1) in hTERT-MSCs under osteogenic conditions [21]. Further observation showed that EVs from the syngenic murine model of MM blocked OBs differentiation and their functionality in vitro [23]. In particular, the authors found that EVs contain and transfer the inhibitor of Wnt/β-catenin pathway, Dickkopf-1 (DKK1) [28], to OBs, leading to the reduction of the OB marker Runx2, Osterix, and Collagen 1A1 [23]. The EV-mediated decrease of Runx2 expression in hMSCs was also correlated to the transport of exosomal antisense lncRUNX2 from MM cells to recipient hMSCs [27].

Specificity Protein 1 (Sp1) is a key transcription factor involved in several biological pathways, acting as positive or negative gene expression regulator; in particular it plays a critical role in cell proliferation, cell cycle progression and apoptosis [29,30,31]. Emerging studies report a role of Sp1 in OB differentiation; in fact, its downregulation prevents vascular calcification by inhibiting the transdifferentiation of vascular smooth muscle cells into OBs [32]. In addition, Sp1 increases the mineralization of osteoblastic cells [33]. Modulation of Sp1 by EVs was recently correlated to their content; in particular Wang and colleagues reported that TGF-β1 increases miR-135 levels in MSC-EVs. EVs containing miR-135 were able to promote chondrocyte proliferation by down-regulating Sp1 [34].

Although different mediators of the inhibitory effect of MM EVs have been already identified, the rich and heterogeneous content of EVs deserves further investigation in order to deeply investigate how MM cell-derivedEVs may act on osteoblast differentiation. In particular, this study aims to correlate the EV-mediated osteogenic inhibition of hMSCs with vesicle content, focusing on miRNAs.

## 2. Results

### 2.1. MM EVs Are Internalized by hMSCs and Inhibit Their Osteogenic Differentiation

**Figure 1:**
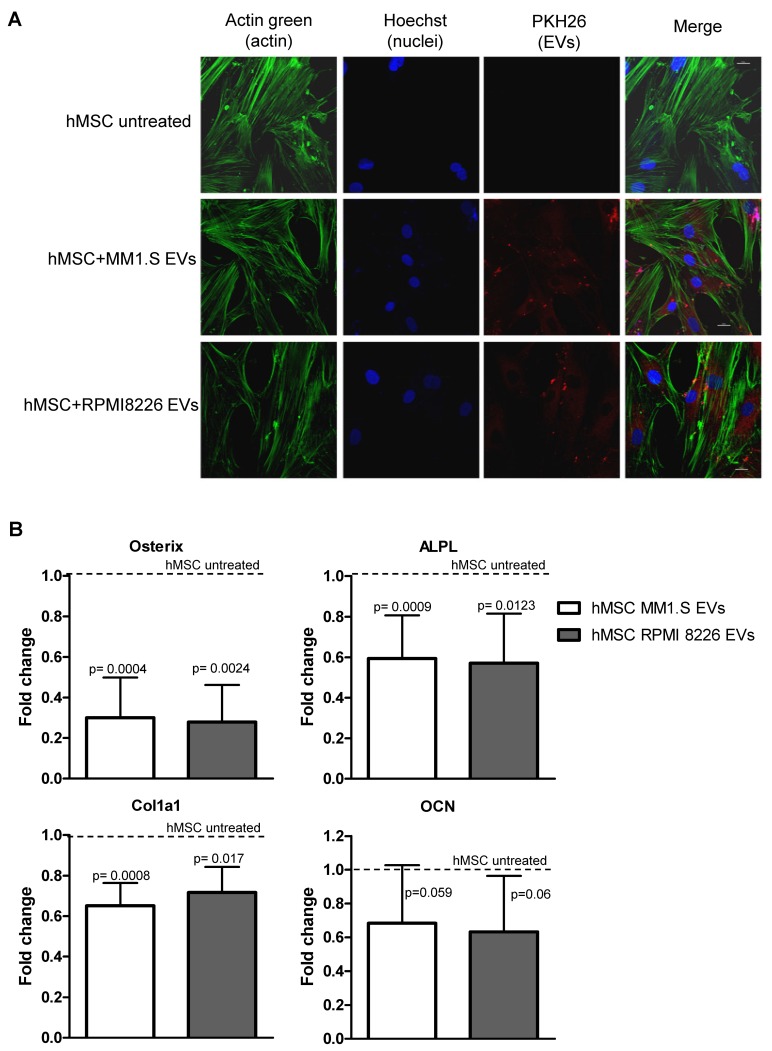
(**A**) Cellular internalization of MM1.S and RPMI 8226-derived EVs into hMSCs was analyzed by confocal microscopy. HMSCs were incubated for 4 h with MM-EVs labeled with PKH26 (red). HMSCs were stained with ActinGreen (green), nuclear counterstaining was performed using Hoescht (blue). Samples were analyzed at the confocal microscope (scale bar = 20µm). (**B**) The Real-Time PCR analyses of Osterix, ALPL, Col1A1 and OCN were performed on hMSCs maintained in the osteogenic medium after 10 days of treatment with MM1.S and RMPI 8226-derived EVs. Data were normalized for β-actin and values are expressed as Fold Change in gene expression that occurred in cells treated with EVs versus untreated hMSCs (dotted line). The statistical significance of the differences was analyzed using a two-tailed Student’s *t*-test.

EVs released by MM cells have been recently correlated to the MM bone disease by directly activating osteoclast functions and, at the same time, by inhibiting the osteogenic differentiation of hMSCs [21,22,23,24,25,27]. In our previous study, we demonstrated that MM EVs inhibit the osteoblastic differentiation of hTERT-MSCs [21]; here we tried to shed light on the mechanism underlying these effects. We first confirmed that EVs from MM1.S and RPMI 8226 cells were internalized by hMSCs (Figure 1A). Then, in order to investigate the effects induced by EVs in hMSCs during osteogenic differentiation, we analyzed the mRNA levels of the early osteogenic differentiation markers, i.e., Osterix, ALPL, Col1A1, and Osteocalcin (OCN), in hMSCs treated for 10 days with EVs from MM cell lines. As reported in Figure 1B, we found a significant and similar reduction of the early markers Osterix, ALPL and Col1A1, between EVs released from the two cell lines, a variable diminished level of Osteocalcin, but we didn’t observe any change in the expression level of Osteopontin, a late osteogenic marker (data not shown). Taken together these results confirmed the role of MM-EVs in inhibiting the early commitment of hMSCs toward osteogenic differentiation.

### 2.2. MM-EVs Contain miRNAs Involved in hMSCsOsteogenic Inhibition

**Figure 2:**
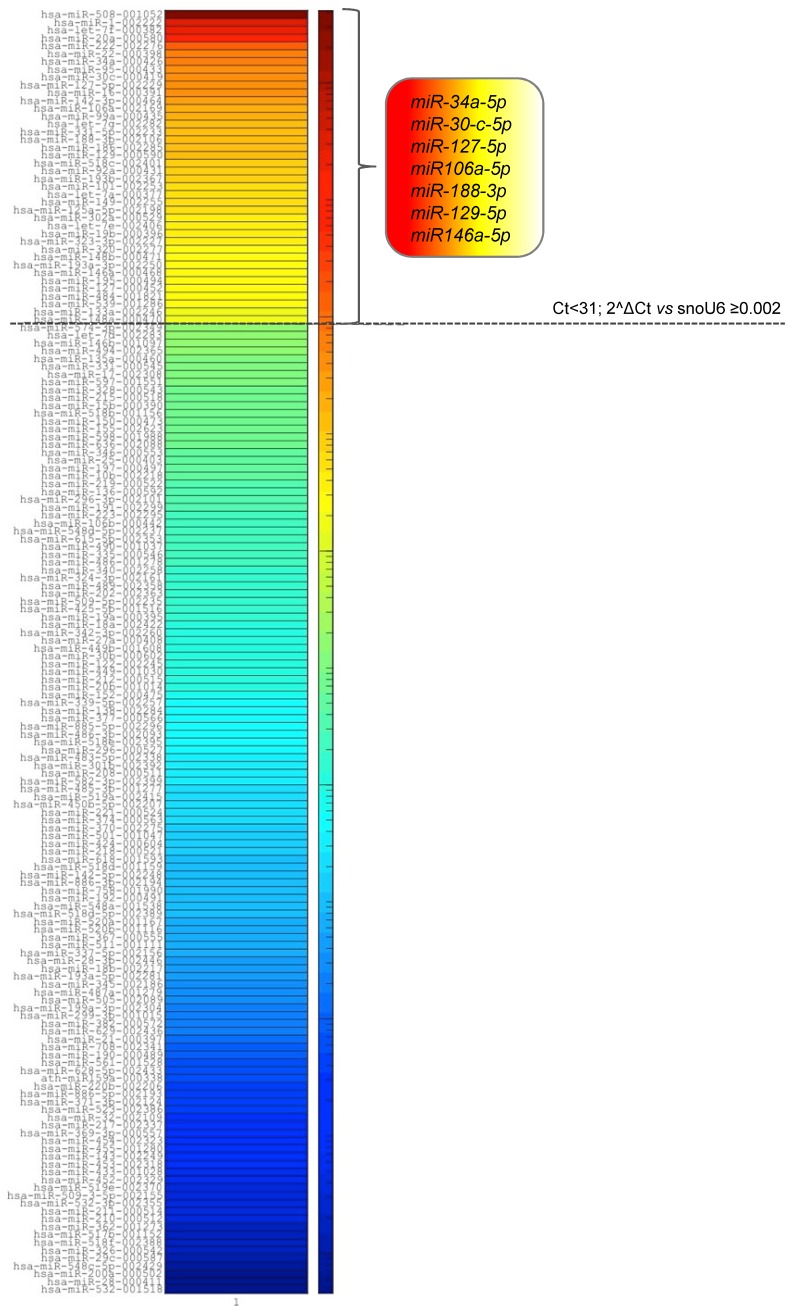
Heat map diagram of the miRNA profile of MM1.S-EVs based on miRNA expression (2^ΔCt vs snoU6). The dotted line separates the miRNAs with higher abundance (Ct <31, 2^ΔCt ≥0.002) from the less abundant species (Ct >31, 2^ΔCt <0.002).

To investigate a possible correlation between the EV-mediated osteogenic inhibition of hMSCs and the miRNA vesicle content, we determined the miRNA expression profile of MM1.S EVs, using a quantitative TaqMan real-time PCR-based microRNA array card. Among the 384 microRNAs contained in the card, we identified 164 miRNAs in EVs from the MM1.S cell line and we then selected the most enriched ones based on their expression level (Ct <31, 2^ΔCt ≥0.002). Out of forty enriched miRNAs, seven of them have been previously correlated with osteogenesis regulation (hsa-miR-34a [35,36,37], hsa-miR-30c [26,38], hsa-miR-127-5p [39,40], hsa-miR-106a [41], hsa-miR-188-3p [37], hsa-miR-129-5p [42,43,44,45], hsa-miR-146a [46,47] (Figure 2)).

**Figure 3:**
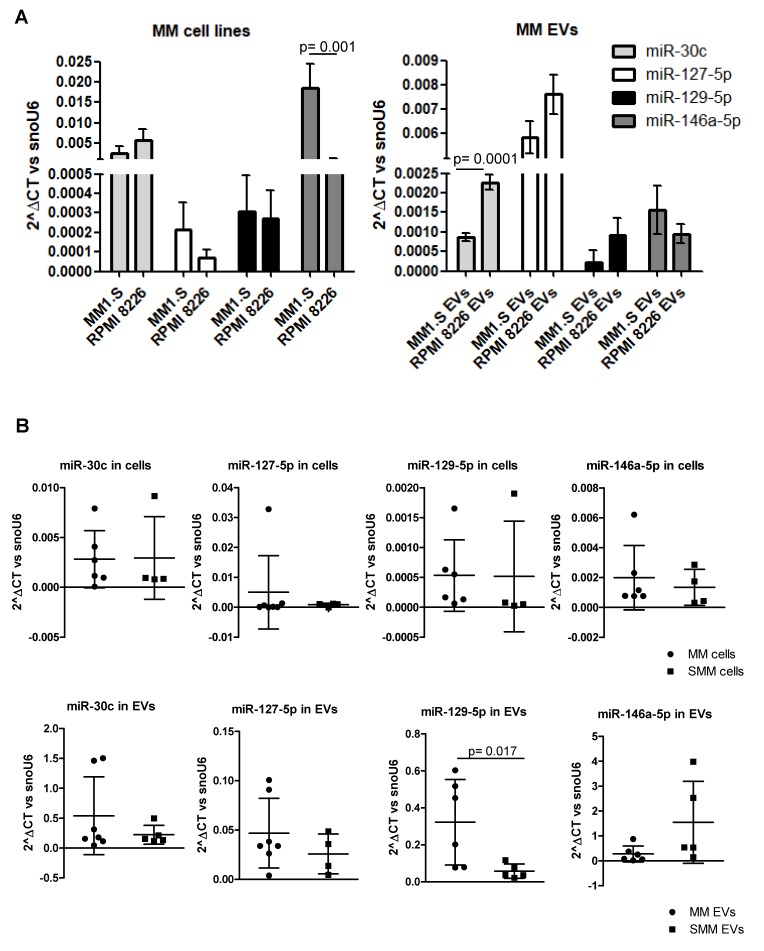
(**A**) Real-time PCR analyses for miR-30c, miR-127-5p, miR-129-5p and miR-146a-5p was performed on MM1.S and RPMI 8226 cells and EVs. The statistical significance of the differences was analyzed using a two-tailed Student’s *t*-test. (**B**) Real-time PCR analysis for miR-30c, miR-127-5p, miR-129-5p and miR-146a-5p was performed on cells and EVs from the bone marrow aspirates of MM patients (n = 6to7) and SMM patients (n = 4to5). Values are plotted as 2^ΔCt versus snoU6. The statistical significance of the differences was analyzed using Mann-Whitney test.

Among those, we decided to focus our attention on hsa-miR-30c, hsa-miR-127-5p, hsa-miR-129-5p, and hsa-miR-146a since their validated and predicted targets are osteoblast differentiation markers. The presence of the four selected miRNAs in MM EVs was further confirmed by qRT-PCR in MM1.S and RPMI 8226 EVs as well as in the respective producing cell lines (Figure 3A). Although each of the four miRNAs is expressed in the two cell lines and is contained in their respective vesicles, we compared the abundance of each miRNA among the two MM cell lines and among the two EV population; to note we observed a comparable amount of miR-30c, miR-127-5p, and miR-129-5p in the two cell lines, while miR146a-5p is more abundant in MM1.S cells compared with RPMI8226. We performed the same analysis on EVs and we found that miR-30c is more abundant in RPMI8226-EVs compared with MM1.S-EVs, while the levels of the other miRNAs was comparable among the two vesicle types.

Once we identified and selected specific microRNAs, we evaluated and compared their abundance in EVs and cells from the bone marrow aspirate samples of patients at different stages of the disease (MM and SMM). As shown in Figure 3B, we did not observe differences in the level of these miRNAs in cells from the two clinical conditions (upper panel), while we interestingly observed a significant enrichment of miR-129-5p in MM EVs compared to SMM (lower panel). Interestingly, two patients with extensive bone disease release EVs with higher miR-129-5p levels. Overall this result may suggest a selective packaging of miR-129-5p into MM-EVs correlated with pathological grade. The clinical characteristics of patients enrolled in this study are summarized in Supplementary Table S1.

### 2.3. MiR-129-5p Level Increases in hMSCs afterEV Incubation

**Figure 4:**
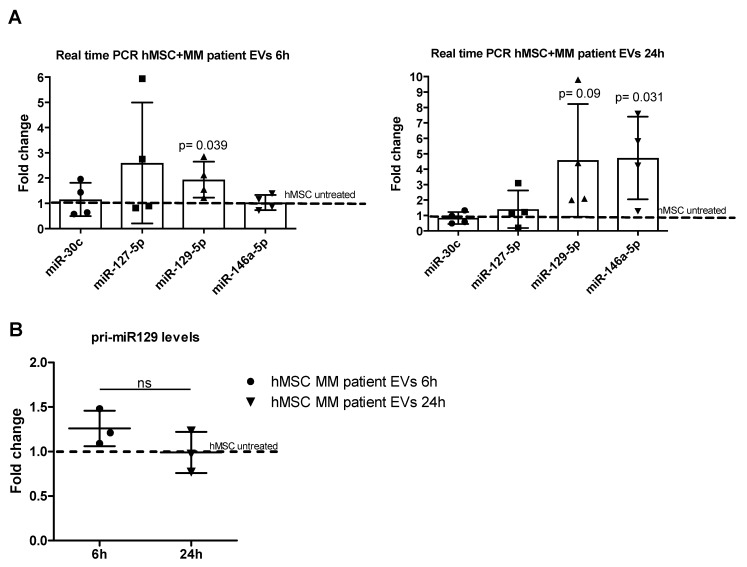
(**A**) Real-time PCR analyses for miR-30c, miR-127-5p, miR-129-5p and miR-146a-5p were performed on hMSCs incubated for 6 (left panel) and 24h (right panel) with EVs from the bone marrow plasma of MM patients (n = 4). Data were normalized for snoU6. Values are expressed as Fold Change in miRNA expression that occurred in cells treated with EVs versus untreated hMSCs (dotted line). (**B**) The Real-Time PCR analyses of pri-miR-129 were performed on hMSCs incubated for 6 and 24h with EVs from the bone marrow plasma of MM patients (n = 3). Data were normalized for β-actin and values are expressed as Fold Change in the pri-miRNA expression that occurred in cells treated with EVs versus untreated hMSCs. The statistical significance of the differences was analyzed using a two-tailed Student’s *t*-test.

Considering that, unlike MM, SMM patients do not exhibit lytic bone lesions, in order to identify the miRNAs that could affect bone differentiation, we focused our study on the miRNAs enriched in EV from MM patients. We first assessed the expression levels of these miRNAs in hMSCs after 6 and 24h of treatment with MM patient EVs (n = 4). To note, three out four EV samples were isolated from patients with extensive bone disease. Interestingly, we found a time-dependent increase of miR-129-5p levels and an increase of miR-146a-5p at 24h (Figure 4A). MiR-146a-5p was previously found in MM EVs and its level increased in MSC after EV treatment. Further, the authors found that the increased level of this miRNA in MSCs led to MM cell growth and migration [48]. Considering the significant increased levels of miR-129-5p among MM EVs (Figure 3B, lower panel) and the increased amount of the miRNA in hMSCs after EV incubation, we decided to focus on miR-129-5p. To correlate the increased level of the miRNA in target cells with its delivery by EVs, we analyzed the miRNA precursor level. As shown in Figure 4B, we didn’t observe any difference in the expression of the pri-miR-129, thus suggesting an EV-mediated transport of the miRNA from MM cells to hMSCs rather than an EV-mediated induction.

### 2.4. MiR-129-5p Inhibits ALPL Expression in hMSCs

**Figure 5:**
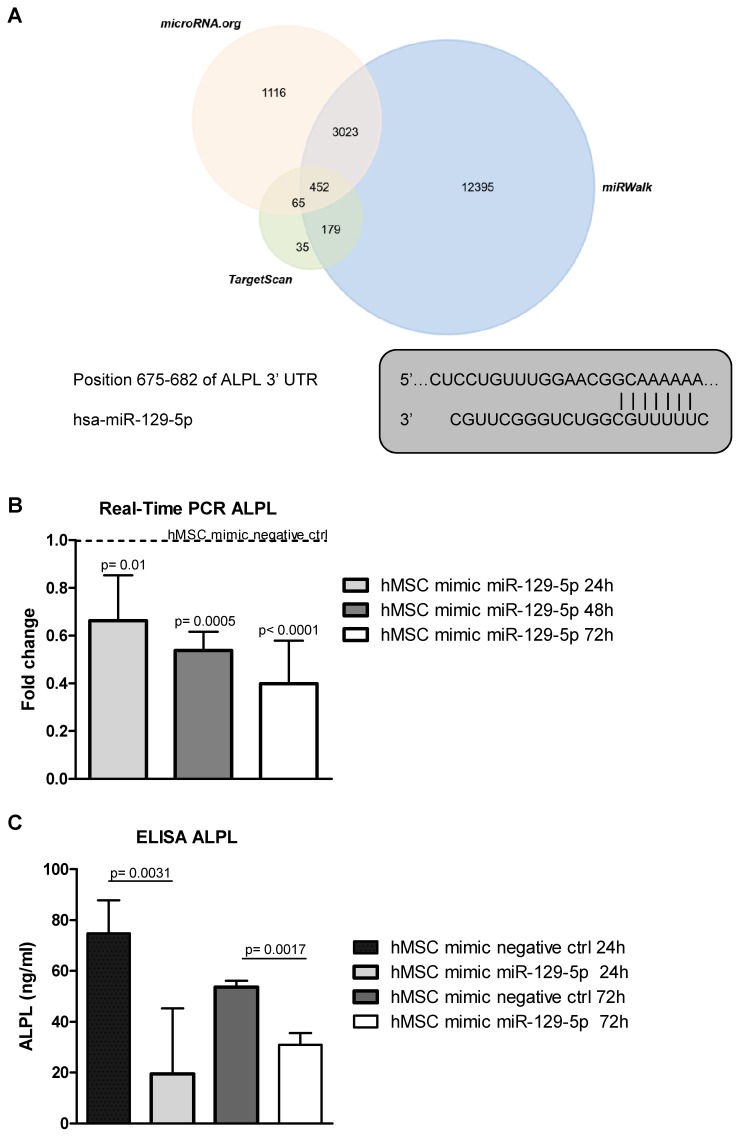
(**A, upper panel**) The target genes of hsa-miR-129-5p were predicted using three different bioinformatics databases (Target scan, miRWalk and microRNA.org). TheVenn diagram was generated using the FunRich tool available online. (**A, lower panel**) The sequence alignment between miR-129-5p and the 3′-UTR of ALPL mRNA predicted by TargetScan. (**B**) The Real-time PCR analysis of ALPL in hMSCs transfected for 24, 48 and 72h with miR-129-5p mimic. Data were normalized for β-actin. Values are expressed as Fold Change in gene expression that occurred in cells transfected with miR-129-5p mimic *versus* hMSCs transfected with mimic negative ctrl for each time point (dotted line). The statistical significance of the differences was analyzed using a two-tailed Student’s *t*-test. (**C**) ALPL release was quantified by ELISA in the supernatant of hMSCs transfected for 24 and 72h with miR-129-5p mimic and with mimic negative ctrl. Values are reported as ALPL concentration in ng/mL. The statistical significance of the differences was analyzed using a two-tailed Student’s *t*-test.

Three publicly available bioinformatics databases (TargetScan, microRNA.org, and miRWalk) were used to analyze genes targeted by miR-129-5p (Figure 5A, upper panel). Among the miRNA predicted targets, we found ALPL (Figure 5A, lower panel), an early marker of hMSCsosteogenic differentiation, whose down-regulation was largely associated with MM EVs-mediated osteogenic inhibition [21,23,24]. To investigate a possible role of miR-129-5p on the EV-mediated osteogenic inhibition of hMSCs, and in particular, on ALPL expression, we transfected hMSCs with the miRNA mimic (Supplementary Figure S1) and subsequently analyzed ALPL levels. We found a reduction of the expression of ALPL both at the mRNA (Figure 5B) and the protein level (Figure 5C).

### 2.5. MiR-129-5p and MM EVs Inhibit the Transcription Factor SP1 Expression in hMSCs

**Figure 6:**
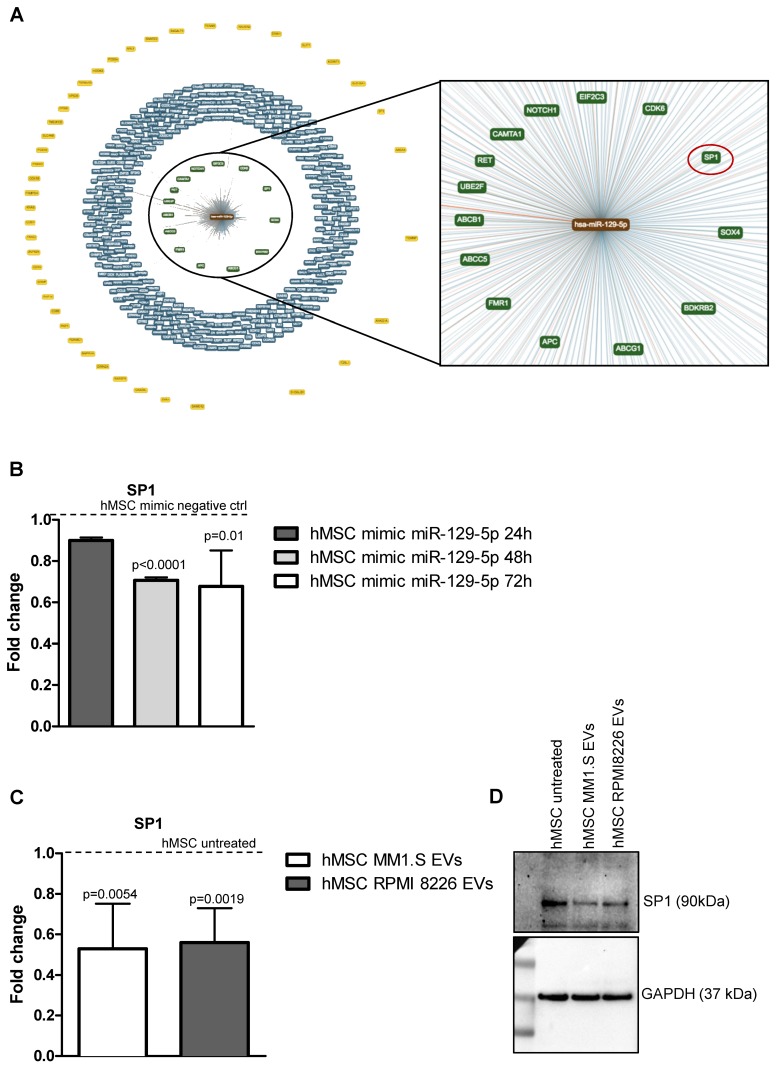
(**A**) The target and predicted genes of hsa-miR-129-5p were analyzed by the bioinformatics tool miRTargetLink Human. The central node represents miR-129-5p, surrounded by the validated target with strong (green) and weak (blue) evidence and the predicted targets in yellow. (**B**) The Real-time PCR analysis of Sp1 in hMSCs transfected for 24, 48 and 72h with miR-129-5p mimic. Data were normalized for β-actin. Values are expressed as Fold Change in gene expression that occurred in cells transfected with miR-129-5p mimic versushMSCs transfected with mimic negative ctrl for each time point (dotted line). The statistical significance of the differences was analyzed using a two-tailed Student’s t-test. (**C**) The Real-time PCR analysis of Sp1 was performed on hMSCs maintained in the osteogenic medium after 10 days of treatment with MM1.S and RMPI 8226-derived EVs. Data were normalized for β-actin and values are expressed as Fold Change in gene expression that occurred in cells treated with EVs versus untreated hMSCs (dotted line). The statistical significance of the differences was analyzed using a two-tailed Student’s *t*-test. (**D**) The expression of SP1 was evaluated by western blotting in hMSCs treated for 10 days with MM1.S and RPMI 8226-derive EVs.

Starting from this data, we next investigated the other targets of miR 129-5p by using the publicly available bioinformatics tool miRTargetLink Human (https://ccb-web.cs.uni-saarland.de/mirtargetlink/) [49]. In Figure 6A, the central node represents miR-129-5p, surrounded by the validated targets with strong (e.g., luciferase assay in green) and weak (e.g., microarray in blue) evidence and the predicted targets in yellow. Among the validated target with strong evidence (Figure 6A, right panel), we found the transcription factor Sp1, largely described as a positive modulator of OB differentiation [33,50,51,52]; it cooperates with Osterix [51,53], and is a transcriptional enhancer of ALPL. Several studies have reported that Sp1 expression is downregulated in cells overexpressing miR-129-5p [54,55]. Therefore, we evaluated Sp1 expression in hMSCs transfected with miR-129-5p mimic and we found that, also in hMSCs, Sp1 is downregulated by miR-129-5p overexpression (Figure 6B). According to this evidence, and to the described role of Sp1 as a positive modulator of osteogenic differentiation of hMSCs, we evaluated the transcription factor levels in hMSCs treated with EVs from MM patients. We observed that Sp1 expression is reduced in target cells treated for 10 days with EVs, both at mRNA (Figure 6C) and protein (Figure 6D) level. Overall, these data indicated in Sp1 modulation a new mechanism by which MM EVs may inhibit hMSCs osteogenic differentiation.

**Figure 7:**
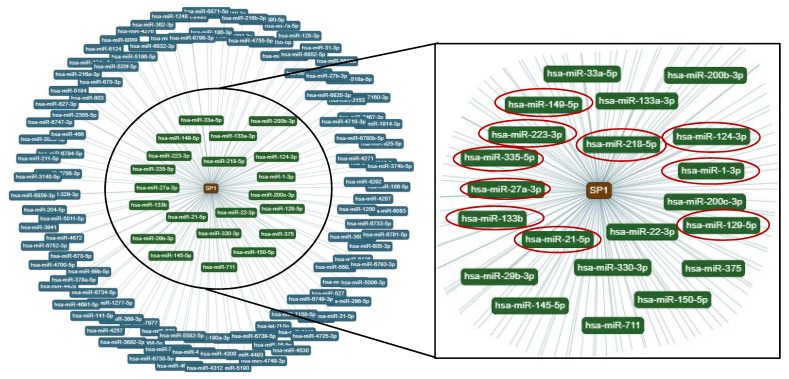
The miRNAs that target Sp1 were analyzed by the bioinformatics tool miRTargetLink Human. The central node represents Sp1, surrounded by the miRNAs that target Sp1 with strong (**green**) and weak (**blue**) evidence. Red circles indicated miRNAs that target Sp1 and that were identified in MM1.S-EVs.

Interestingly, a bioinformatics analysis, focusing this time on the transcription factor Sp1 (central node in Figure 7, left panel), revealed that among the miRNAs that target Sp1 with strong evidence, the 50% were identified by us in MM1.S EVs by TaqMan real-time PCR-based miRNA array card (Figure 7, right panel, red circle). Altogether these data suggest that several mediators of the osteogenic differentiation of hMSCs are modulated by MM EVs and that, together with miR-129-5p, other miRNAs, delivered by EVs may cooperate to decrease the osteogenic potential of hMSCs.

## 3. Discussions

Multiple lines of evidence define the involvement of EVs in MM progression as they can act on several cellular targets and at different levels of disease progression [12,13,14,15,16,17,18,19]. In particular, recent findings have highlighted the role of EVs as inducers of MM-osteolytic bone disease [21,22,23,24,25,26,27].

Although some of the mediators of this effect have been identified, a further description of the content of the EVs and the identification of additional molecules responsible for the EV-mediated activation of OCs and the inhibition of OBs would be useful to develop new diagnostic and prognostic markers in MM management. In the current study, we assess if miRNAs transported by EVs may have a role in the inhibition of osteoblast differentiation.

OBs are derived from bone marrow MSCinduced to differentiate bymicro-environmental cues represented by cytokines and extracellular matrix components [56]. In MM patients, the osteogenic ability of mesenchymal progenitors to differentiate to mature OBs is disrupted [57,58,59], as evidenced by the decrease in the levels of Osterix, the major osteoblast transcriptional activator, and of the bone formation markers, i.e., ALPL and Collagen type I α1 chain [60,61,62]. In this context, we found that MM-EVs strongly downregulate the expression of these three markers actively participating in the inhibition of the first differentiation steps of mesenchymal cells.

It is already well known that EVs contain several species of non-coding RNAs including miRNAs that can be carried and transferred to target cells, thus affecting their phenotype [63]. Here, we found that many of the miRNAs identified in MM EVs have been previouslycorrelated to the inhibition of osteogenic differentiation markers and that the levels of the miR-129-5p are correlated with the pathological grade of MM. Of note, miR-129-5p contains the hEXO motif, a short sequence previously identified by Santangelo et al. which is recognized by the RNA-Binding Protein SYNCRIP, leading to the exosomal enrichment of some miRNAs [64]. This observation enforces the hypothesis of a selective packaging for miR-129-5p.

A very interesting aspect emerging from the analysis on patient samples is that, since the occurrence of MM is often preceded by the asymptomatic plasma cell proliferative disorder (SMM) [65], vesicle-associated miR-129-5p may represent a new biomarker for the risk stratification; further studies, as well as the increase in the number of enrolled patients, are needed to validate this point.

Concomitantly, by mimic transfection, we investigated the effect of miR-129-5p on hMSCs by focusing on the predicted and validated osteogenic markers. MiR-129-5p was found to bind to the 3′UTR of COL1A1 mRNA in gastric cancer [42,44] and hepatic stellate cells [43]. Consistent with these findings, we observed a downregulation of COL1A1 mRNA in hMSCs transfected with miR129-5p mimics (data not shown). The decrease in ALPL, induced by EVs, and specifically by miR-129-5p, supports the role of EV-miRNAs in MM bone disease.In fact, alkaline phosphatase is decreased in the serum of patients with MM bone involvement [66], while an increase in its level, in patients treated with bortezomib, was associated with treatment response [67].

Once in hMSCs, miR-129-5p can affect cell phenotype by targeting different transcripts; bioinformatics analyses revealed that several validated targets of miR-129-5p are involved in bone remodeling pathways, such as the ATP-binding cassette transporters [68,69] and Notch1 [70,71]. Of note, among the validated targets, we found the transcription factor Sp1 [54,55]. Sp1 was previously described as a positive regulator of OB differentiation [33,50,51,52], in fact, its downregulation in a human fetal osteoblastic cell line (hFOB) resulted in reduced expression of Frizzled-1 and a decrease in ALP activity, while its overexpression increased osteosarcoma cell Saos2 mineralization [33]. Further, Sp1enhances Runx2 promoter activity during osteogenesis progression [72].

Our study highlighted a novel mechanism by which MM EVs can inhibit osteoblast differentiation, by reducing the expression of Sp1 in hMSCs. Interestingly, focusing on the validated miRNAs that target Sp1, half of them are present in MM-EVs and are known to have a role in osteogenic differentiation of MSC. For example, miR-21-5p inhibited the osteogenic differentiation in Periodontal Ligament Stem Cells by targeting Smad5 [73]. In addition, its presence in osteosarcoma exosomes was correlated with bone resorption activity of pre-osteoclast cells. The miR-27a-3p expression is upregulated in osteosarcoma cell lines compared with normal human osteoblastic cells [74]. The evidence that different molecular players, with the same target, are delivered by EVs, emphasizes the ability of EVs to strongly affect the phenotype of recipient cells. Further investigation are needed to deeply investigate the in vivo contribution of Sp1 in the osteogenic differentiation of MSC in patient affected by MM. At this stage, consistent with previous data, we correlated the MM-EV miRNA content with the dimished Sp1 levels in hMSC. To date, Sp1 expression was correlated to MM cell proliferation [75,76] but its expression in hMSC during MM bone disease development still need to be explored.

In conclusion, our study provides new insights into the role of MM EVs in inhibiting the osteogenic differentiation of hMSCs focusing initially on the content of EVs. Then, taking into consideration the increase in miR-129 levels in hMSCs after EV treatment, we unveiled a novel mechanism, by which MM EVs may act on osteoblast differentiation.

## 4. Materials and Methods

### 4.1. Cell Cultures

MM cell lines, MM1.S and RPMI 8226 were purchased from ATCC (Manassas, VA, USA) and cultured in RPMI 1640 medium (Euroclone, UK) supplemented with 10% fetal bovine serum (FBS, Euroclone s.p.a., Milan, Italy). FBS was previously ultracentrifuged to deplete it from exosomes (exosome- free FBS). Human Mesenchymal Stem Cells (hMSCs) were obtained from Lonza (Lonza, Walkersville, MD, USA) and grown in Mesenchymal Stem Cell growth medium (MSCGM BulletKit™, Lonza, Walkersville, MD, USA) to maintain them into an undifferentiated condition and in Mesenchymal Stem Cell Osteogenic Differentiation Medium to induce osteogenic differentiation (MSC Osteogenic Differentiation BulletKit™, Lonza). Cells were routinely tested for mycoplasma contamination using N-Garde Kit For Mycoplasma Detection (Euroclone).

### 4.2. Purification of Primary Plasma Cells

Human primary plasma cells were isolated from the bone marrow aspirates of patients affected by MM patients (n = 7) and by Smouldering Multiple Myeloma (SMM, n = 5) in accordance with the Declaration of Helsinki guidelines and the Ethics committee of the Hospital of the University of Palermo (date of approval 14/11/2018, report N°10/2018). Bone marrow blood sampleswere diluted with PBS and mononuclear cells were separated by Ficoll-Paque gradient centrifugation (GE Helthcare-Bio Science, Uppsala, Sweden). The collected cells were pelleted and stored for further RNA isolation.

### 4.3. Extracellular Vesicle Isolation

Extracellular vesicles (EVs) were isolated from the conditioned culture medium of MM1.S and RPMI 8226 after a 48h culture period in the presence of exosome- free FBS. EVs were collected by differential centrifugation; conditioned culture medium was centrifuged subsequently for 5 min at 300× *g*, 15 min at 3000× *g*, and 30 min at 10,000× *g* and then ultracentrifuged for 105 min at 100,000× *g* in a Type 70 Ti, fixed angle rotor. EVs were also isolated from the bone marrow (BM) plasma of patients affected by MM (n = 7) and by SMM (n = 5). All patients provided written informed consent in accordance with the Declaration of Helsinki. The Ethics Committee of the Hospital of the University of Palermo (Italy) approved the study. EVs were isolated from human plasma and prepared as described above. EV pellets were washed and suspended in PBS, and vesicle protein content was determined by the Bradford assay.

### 4.4. Internalization of MM-Derived EVs by hMSCs

MM1.S and RPMI 8226 EVs were labeled with PKH26 Red Fluorescent Cell Linker Kits (Sigma–Aldrich, USA) following the datasheet information. EVs, collected as previously described, were incubated for 15 min at room temperature with PKH26 dye previously diluted in the diluent C solution. Labeled EVs were washed in PBS; the pellets were suspended in the medium and incubated with hMSCs, grown on coverslips, for 4 h. After incubation, hMSCs were fixed with PFA 4%, permeabilized with 0.1%TritonX-100, stained with Actin Green (Molecular Probes, Life Technologies, Carlsbad, CA, USA) that binds actin with high affinity. Nuclei were then stained with Hoechst (Molecular Probes, Life Technologies, Carlsbad, CA, USA). Preparations were analyzed by confocal microscopy (Nikon A1).

### 4.5. hMSCs Treatment with EVs

Cells were plated in 12-well plates in the basal Mesenchymal Stem Cell growth medium; 24 h after seeding, when cells were 70% confluent, the media was replaced with the Mesenchymal Stem Cell Osteogenic Differentiation Medium. Cells were treated for 10 days with EVs from MM1.S and RPMI 8226 (50 μg/mL) to assess the effect of EVs on the osteogenic differentiation of hMSCs; the media were changed every 3 days. Cells were also treated for 6 and 24h with EVs from plasma samples to further evaluate the miRNA expression levels.

### 4.6. MiRNA Expression Profiling

RNA was extracted from MM1.S EVs by using the IllustraRNAspin Mini Isolation Kit (GE Healthcare, Little Chalfont, Buckinghamshire, UK) according to the manufacturer’s recommendations. 200 ng of total RNA was reverse transcribed using Megaplex™ RT Primers Human Pool A (Life Technologies, Carlsbad, CA, USA) according to the manufacturer’s instructions. The reaction was carried out at 16 °C for 2 min, 42 °C for 1 min, 50 °C for 1 s for 40 cycles, 85 °C for 5 min then hold at 4 °C.

To identify the miRNA profile of MM1.S EVs, real-time TaqMan PCR was carried out by using the TaqMan microRNA array Human Pool A cards containing 384 different miRNAs (Thermo Fisher Scientific, Waltham, MA, USA). The cDNA obtained in the previous step was diluted, mixed with TaqMan Gene Expression Master Mix and loaded into each of the eight fill ports on the TaqMan^®^ Human MicroRNA Array A (Life Technologies, Carlsbad, CA, USA). The array was centrifuged twice at 1200 rpm for 1 min and run on ABI-PRISM 7900 HT Sequence Detection System (Applied Biosystems, Foster City, CA, USA) using the manufacturer’s recommended program. Ct values for each miRNA were normalized using thereferencesnoU6 RNA. Relative miRNA levels were expressed as 2^ΔCt. The HeatMap has been generated using Matlab 2019a software. Color scaling is set to log scale.

### 4.7. Cell Transfection

Transfection of miRNA mimic in hMSCs was performed using the Lipofectamin 3000 Transfection Reagent (Thermo Fisher Scientific) according to the manufacturer’s instructions. Cells were plated in 12-well plates; 24 h after seeding, when cells were 70% confluent, cells were transfected with 30pmoles/mL of hsa-129-5p mimic (cat.number 4464066, Life Technologies), or scrambled negative control (4464058, Life Technologies). Twenty-four, 48 and 72 h after transfection, the medium was collected and the cells processed for RNA isolation.

### 4.8. RNA Isolation and Real-Time PCR

RNA was extracted using the commercially available IllustraRNAspin Mini Isolation Kit (GE Healthcare, Little Chalfont, Buckinghamshire, UK), according to the manufacturer’s instructions. RNA was isolated from cell lines, primary cells as well as EVs. Total RNA was reverse transcribed to cDNA using the High Capacity cDNA Reverse Transcription Kit (Applied Biosystems, Foster City, CA, USA). For quantitative Sybergreen real-time PCR, the reaction was carried out in a total volume of 20μLcontaining 2× SYBR Green I Master Mix (Applied Biosystems), 2μLcDNA, and 300 nM forward and reverse primers. The oligonucleotides used were reported in the Table 1 below:

For miRNA expression, 10 ng of RNA was reverse transcribed according to the manufacturer’s instructions (TaqManMicroRNA Reverse Transcription, Applied Biosystem). Taqman probes, from Applied Biosystems were used to analyze: miR-30c-5p, miR-127-5p, miR-129-5p, miR-146-5p, and U6. Real-time PCR was performed in 48-well plates using the Step-One Real-Time PCR system (Applied Biosystems) in duplicated for each data point. Relative changes in the target mRNA content, relative to a housekeeping gene (β-actin), and in the target miRNA content, relative to housekeeping U6, were determined with the ΔΔct Method.

### 4.9. ELISA ALPL

ALPL concentration in the conditioned medium of hMSCs was measured using the ALPL ELISA Kit (MyBioSource), following the manufacturer’s protocol. The conditioned medium of hMSCs was medium was collected after 24 or 72h of transfection with miR-129-5p mimic. Data were expressed as ALPL concentration in ng/mL.

### 4.10. Western Blotting

Total proteins from hMSCs cells, treated for 10 days with MM-EVs, were isolated and analyzed by SDS-PAGE followed by western blotting. Antibodies used in the experiments were as follows: anti-SP1 antibody (Abcam) and anti-GAPDH (Santa Cruz Biotechnology, CA, USA).

### 4.11. Bioinformatics Tools

TargetScan [77], miRWalk [78] and microRNA.org [79] bioinformatics databases were used to analyze the predicted target mRNAs of the selected microRNAs. FunRich analysis tool [80] was used to generate a Venn diagram of the relationships between the 3 datasets generated above. miRTargetLink Human [49] was used to generate the miRNA-target interaction network.

### 4.12. Statistical Analysis

Data are reported as mean ± standard deviation (SD) of 3 or more biological replicates. Statistical analysis was performed using GraphPad Prism software (GraphPad software, Inc, La Jolla, CA). The statistical significance of the differences was analyzed using a two-tailed Student’s *t*-test. When the data were not normally distributed, Mann-Whitney test was used. A *p*-value ≤0.05 was considered significant.

### 4.13. Ethical statement:

All subjects gave their informed consent for inclusion before they participated in the study. The study was conducted in accordance with the Declaration of Helsinki; the protocol was approved by the Ethics Committee of the Hospital of the University of Palermo (date of approval 14/11/2018, report N°10/2018). 

## 5. Conclusions

In summary, in this study, we have provided evidence of the role of miRNAs, delivered by MMEVs, on the EV-mediated osteogenic inhibition of hMSCs. In particular, we found that MM-EVs contain several miRNAs involved in osteogenic regulation. Among those, miR-129-5p is enriched in MM EVs and is delivered in hMSCs, leading to the downregulation of ALPL and Sp1 (Graphical abstract). Further, our study uncovered the transcription factor Sp1, already described as a positive modulator of hMSCsosteogenic differentiation, as a novel target of MM EVs.

## Figures and Tables

**Table 1:** Primer sequence used for gene expression analysis.

Gene	Primer Forward	Primer Reverse
B-Actin	TCCCTTGCCATCCTAAAAAGCCACCC	CTGGGCCATTCTTCCTTAGAGAGAAG
OSX	TGCTTGAGGAGGAAGTTCAC	AGGTCACTGCCCACAGAGTA
ALPL	CCACGTCTTCACATTTGGTG	AGACTGCGCCTGGTAGTTGT
COL1A1	TGTGGATGCCTCTTGGGTATC	TTTTGGCCATCTCTTCCTTCA
OCN	AGCAAAGGTGCAGCCTTTGT	GCGCCTGGGTCTCTTCACT
SP1	GCCTCCAGACCATTAACCTCAGT	GCTCCATGATCACCTGGGGCAT

## Supplementary Materials

The following are available online at https://www.mdpi.com/2072-6694/12/2/449/s1, Supplementary Table S1: Clinical characteristics of patients affected by MM and SMM; Supplementary Figure S1: Real-time PCR analysis of miR-129-5p in hMSCs transfected for 24, 48 and 72h with miR-129-5p mimic.
