# Supplementary material for: Extracellular Vesicle microRNAs Contribute to the Osteogenic Inhibition of Mesenchymal Stem Cells in Multiple Myeloma
Source: Cancers (Basel). 2020 Feb 14;12(2):449. doi: 10.3390/cancers12020449 (PMC7072478; doi:10.3390/cancers12020449)
Supplement: Supplementary file 1 [file cancers-12-00449-s001.pdf]

	Isotype	Hemoglobin g/dL	Calcemia mg/dL	LDH (U/L)	Medullary plasma cell infiltrate (%)	M component (g/dL)	Osteolytic lesions	Bone disease extent
**MM Patients**								
MM#1	LCMM λ	10.7	9.65	287	20	17800 mg/L (monoclonal free light chains- micromolecular MM)	absent	
MM#3	MM IgG k	14.7	8.9	199	12	0.54	present	Dorsal and lumbar spine (widespread involvement) - righ ribs (VI and VIII)
MM#4	MM IgA λ	10.2	9.75	183	40	1.27	absent	
MM#5	IgG λ	15	9.63	176	35	1.25	present	Cervical (C7) and dorsal (D11 and D12) spine
MM#6	IgA λ	11.3	9.89	100	40	2.64	absent	
MM#7	IgA λ	11	9.02	149	9	0.91	present	Lumbar spine (L3 and L4)- Right ischio-pubic branch
MM#10	LCMM λ	9	8.61	190	80	1570 mg/L (monoclonal free light chains- micromolecular MM)	absent	
**SMM Patients**								
SMM#2	IgG λ	13.6	8.74	100	20	2.4	absent	
SMM#8	IgG k	11.6	9.57	149	6	2.25	absent	
SMM#13	IgG k	15.7	9.9	148	8	1.79	absent	
SMM#17	IgG k	13.4	9.14	185	40	2.32	absent	
SMM#18	IgG k	12.8	9.96	147	15	1.62	absent	

MM: Multiple Myeloma
LCMM: Light Chain Multiple Myeloma

Supplementary Table S1. Clinical characteristics of patients affected by MM (n=7) and SMM (n=5).

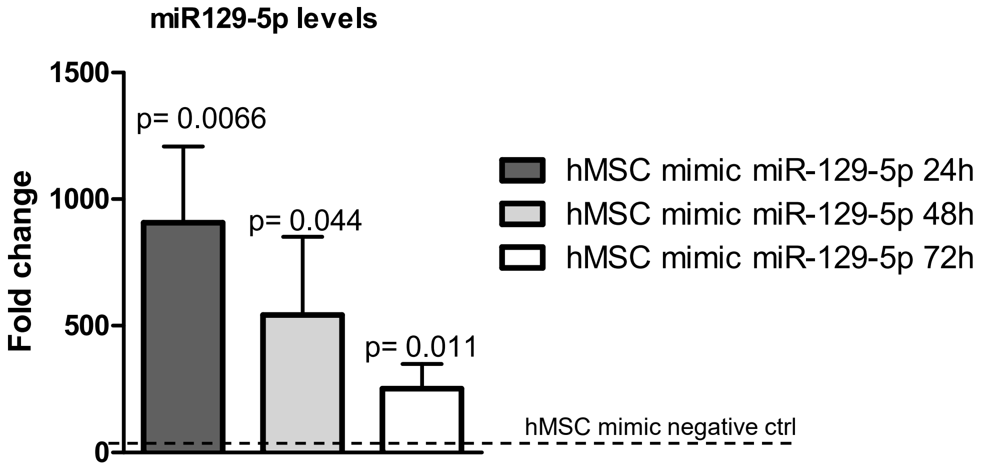

Supplementary Figure S1. Real-time PCR analysis of miR-129-5p in hMSCs transfected for 24, 48 and 72h with miR-129-5p mimic. Data were normalized for snoU6. Values are expressed as Fold Change in microRNA expression that occurred in cells transfected with miR-129-5p mimic versushMSCs transfected with mimic negative ctrl for each time point (dotted line). The statistical significance of the differences was analyzed using a two-tailed Student's t-test.

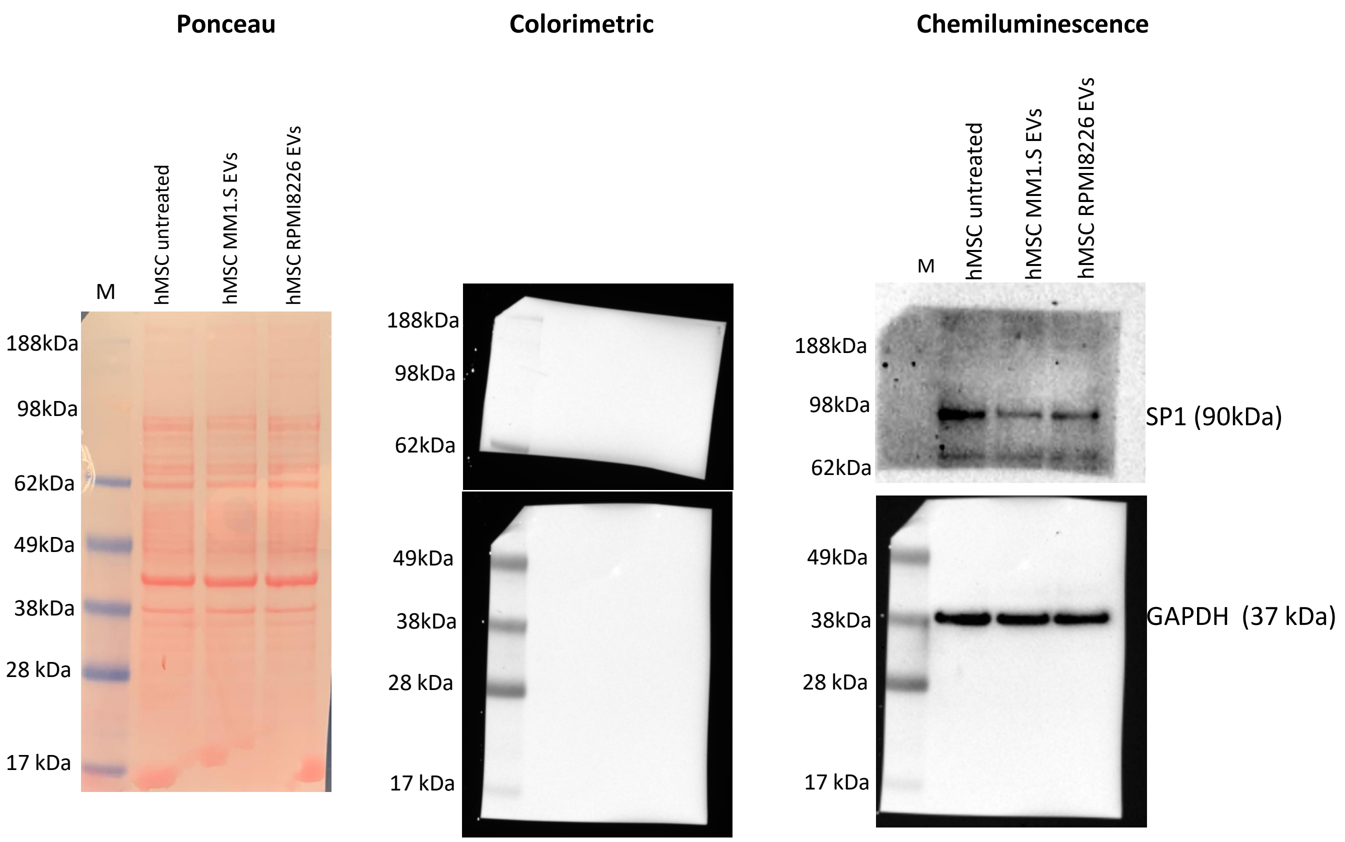

SAMPLES	Volume GAPDH	Volume SP1	SP1/GAPDH	fold change
hMSC untreated	7485880	540046	0,072142	1
hMSC MM1.S EVs	6421098	218933	0,034096	0,47262211
hMSC RPMI8226 EVs	6299422	248267	0,039411	0,54629899

Supplementary Figure S2: Full blot of Figure 6D
